# Unveiling the Enigma: Idiopathic Digital Infarction—A Case Report and Literature Review

**DOI:** 10.1002/ccr3.70107

**Published:** 2025-01-30

**Authors:** Jaber H. Jaradat, Wadi Walid, Aram F. Obeidat, Raghad Amro, Abdulqadir J. Nashwan

**Affiliations:** ^1^ Faculty of Medicine Mutah University Al Karak Jordan; ^2^ Department of Internal Medicine Al‐Karak Governorate Hospital Al‐Karak Jordan; ^3^ Department of Internal Medicine, Faculty of Medicine Mutah University Al‐Karak Jordan; ^4^ Nursing Department Hamad Medical Corporation Doha Qatar; ^5^ Department of Public Health, College of Health Sciences, QU Health Qatar University Doha Qatar

**Keywords:** digital infarction, digital ischemia, hand, idiopathic

## Abstract

Idiopathic digital infarction (IDI), a rare subset of digital infarction, is characterized by ischemic changes in the absence of identifiable underlying etiology. We present the first documented case of IDI in a 47‐year‐old female with insignificant medical history. Clinical evaluation revealed bluish discoloration of the left lateral three fingers. Negative findings on autoimmune serologies, echocardiogram, and coagulation profiles excluded common causes, such as vasculitis, cardiac embolism, and hypercoagulable states. Imaging studies demonstrated ischemic changes in the left cerebellar hemisphere and proximal left subclavian artery, which supported the diagnosis of acute infarction. The patient was managed conservatively and was discharged. This case underscores the diagnostic complexities of IDI and highlights the importance of thorough evaluation and management.

**Summary**

- This case emphasizes the importance of a comprehensive diagnostic approach for assessing patients with digital infarctions.
- Physicians must exclude all identifiable etiologies before concluding idiopathic digital infarction.

## 1 Introduction

Diminished blood perfusion poses a significant threat to tissue viability, progressing from ischemia to eventual tissue death, commonly referred to as infarction. The underlying etiology of ischemia or infarction is diverse, ranging from vasoconstriction or blockage of the supplying vessels to manifestation of systemic diseases such as vasculitis. Digital ischemia results from inadequate blood supply to digital tissue, subsequently leading to digital infarction (DI). The presence of digital pain accompanied by pallor or cyanosis of the skin alarm of impending digital infarction [1]. Idiopathic digital infarction (IDI) is a rare manifestation within the spectrum of digital infarction. Raimbeau et al. estimated the prevalence of IDI is 11.7% [2].

The diagnostic approach for digital infarction commences with a thorough patient history, guiding subsequent investigations, including noninvasive imaging modalities such as Doppler ultrasound (US), angiography, and magnetic resonance angiography (MRA) [1, 3]. Additionally, autoimmune antibodies, such as ANA and ANCA, can provide valuable insights to support diagnosis [4]. However, the diagnosis of idiopathic digital infarction is a diagnosis of exclusion, which is established after other diagnoses have been ruled out.

Effective treatment of digital infarction stems from early diagnosis before tissue viability is lost. Treatment approaches for DI vary from lifestyle changes and avoidance of stressors to medical therapy, such as using vasodilators and vasoprotective drugs [1, 3, 4].

Here, we report the first case report of idiopathic digital infarction in a healthy female. We discussed the possible causes of ID. In this case, the diagnosis was unattainable even after a through examinations, and investigations, and therefore, it is worth reporting, to highlight this rare case, and to encourage future research to identify possible clinical scenarios and underlying causes. We also present a review of the literature over the past 5 years on the causes that might result in digital ischemia/infarction.

## 2 Case History and Examination

**FIGURE 1:**
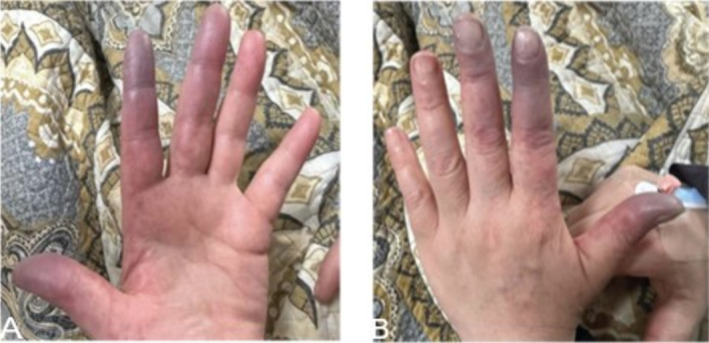
Shows infraction of the lateral three fingers of the left hand. (A) shows the palmar aspect, involving the distal portions of the lateral three fingers, and (B) shows the dorsal aspect the left hand.

A 47‐year‐old female with no remarkable medical history was admitted via the ER complaining of bluish discoloration of her left lateral three fingers that started 3 months prior to admission (Figure 1). Moreover, she confirmed the absence of experiencing Raynaud's phenomenon. On the physical exam, the patient was stable with no remarkable signs of systemic diseases or cardiovascular diseases. Additionally, there was no oral or genital ulcers, and the patient denied the presence of arthralgia, and other full systemic examination from head to toe was not significant. Blood pressure was 125/90, and her other vitals were within normal range.

## 3 Differential Diagnosis and Investigations

**FIGURE 2:**
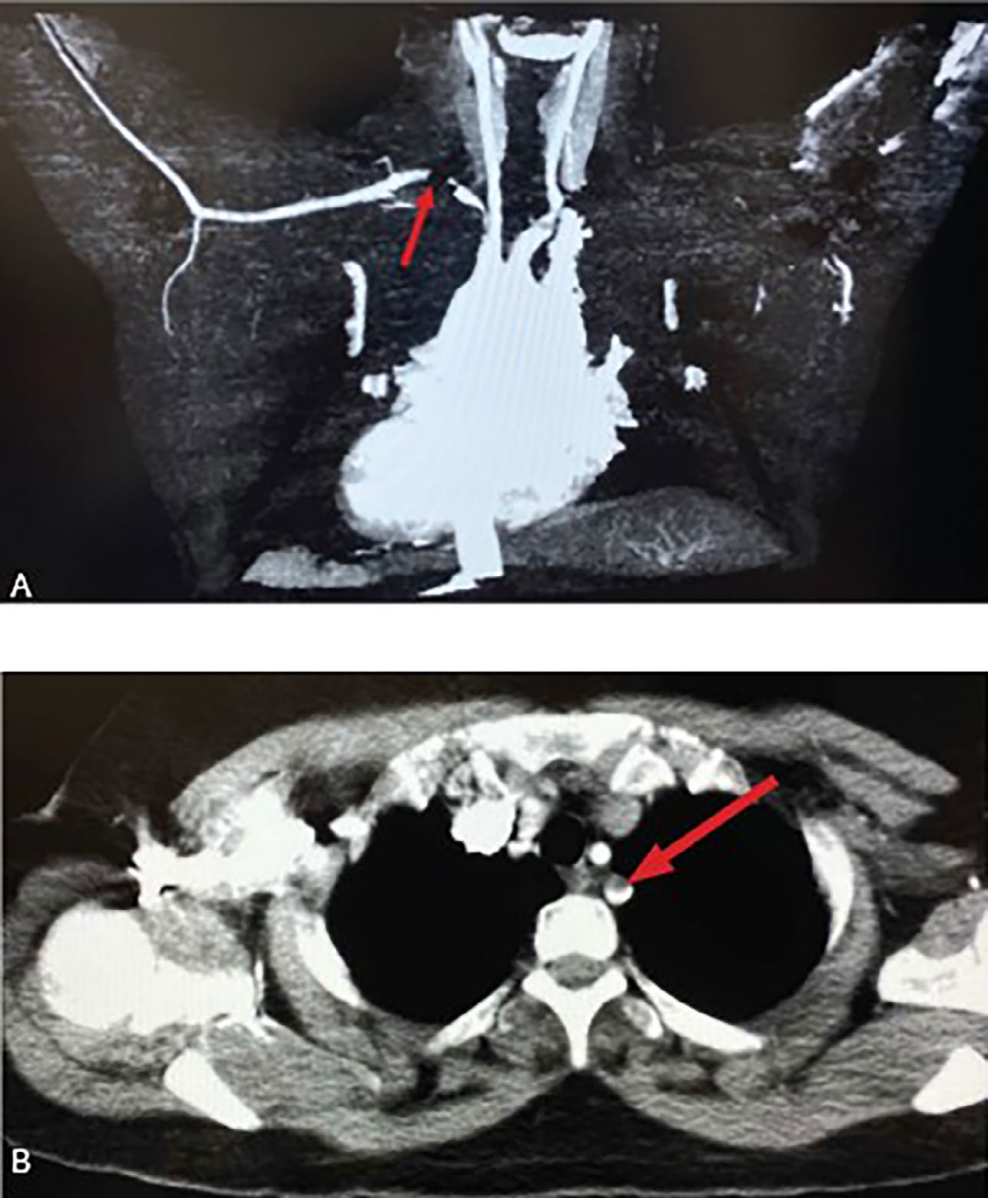
Displays two images obtained through CTA. In images A and B, the arrow points to the partial filling defects are observed in the proximal portion of the left subclavian artery.

**FIGURE 3:**
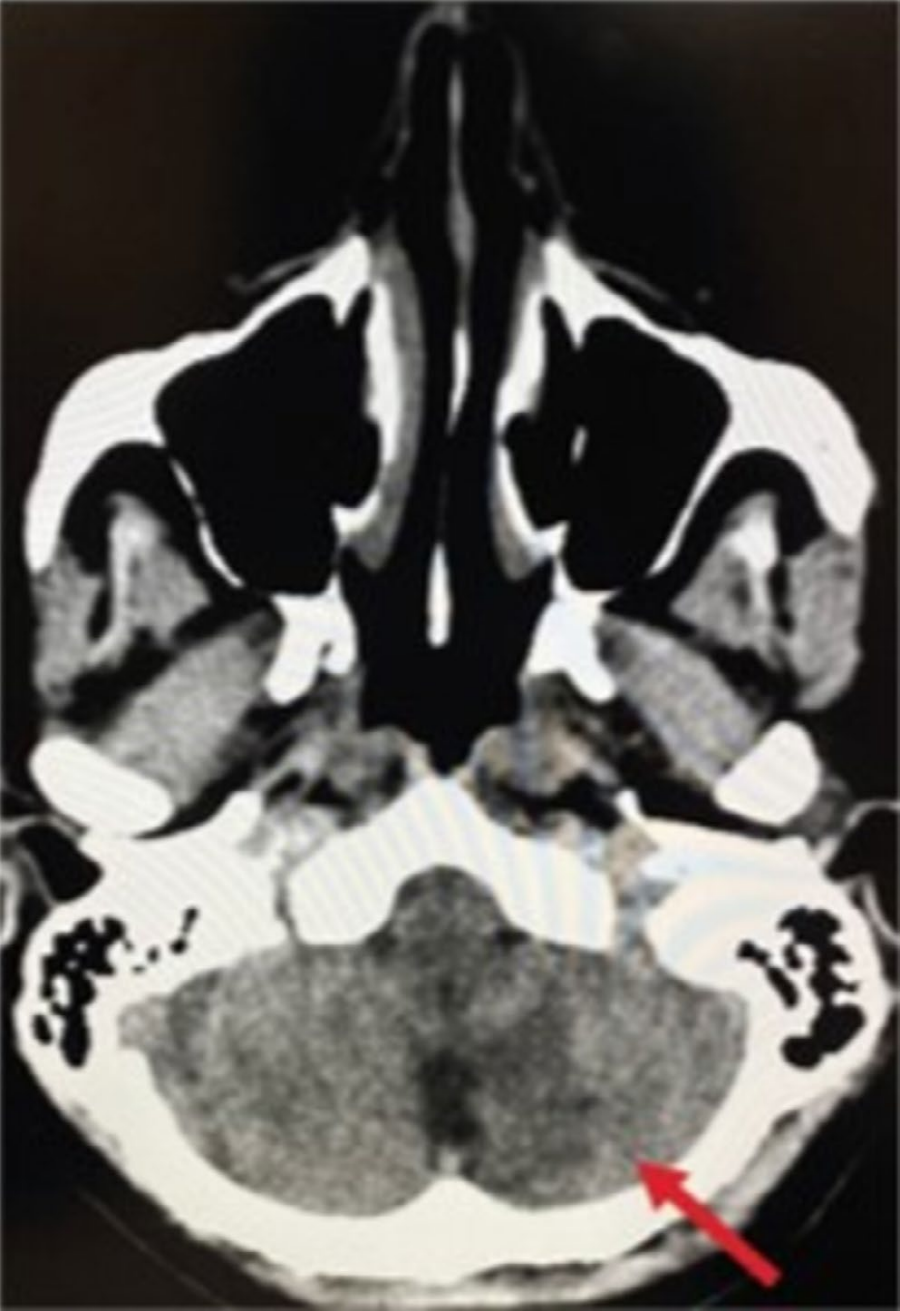
Shows a brain CT scan. The arrow points to subtle hypodensities in the left cerebellar hemisphere. These hypodensities may indicate various underlying conditions such as ischemia, infarction, or other pathological changes.

**FIGURE 4:**
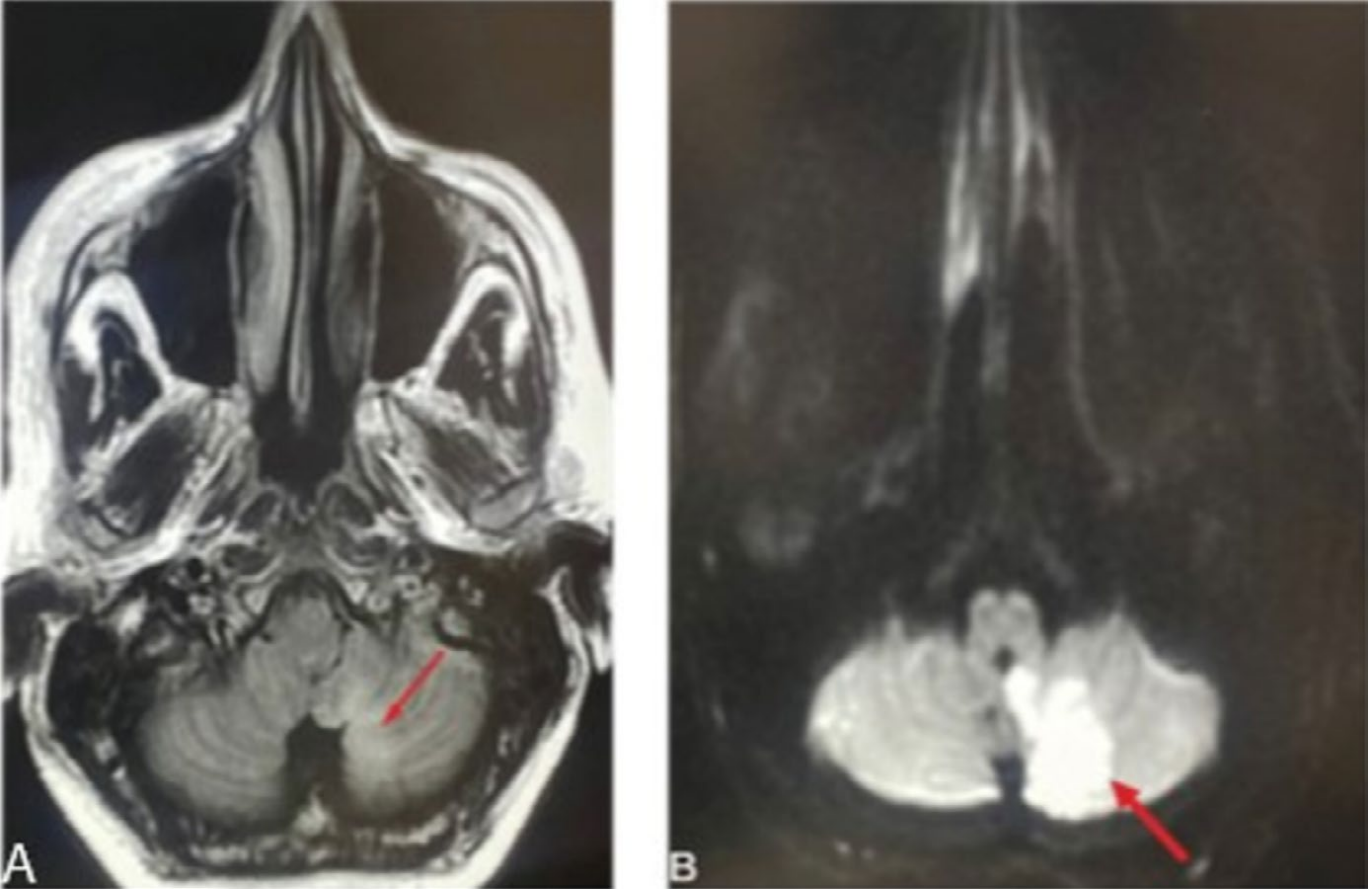
(A) Shows a T1 intensity MRI, and the arrow points hypointensity in the left cerebellar hemisphere, in the inferior medial portion. (B) shows diffusion restriction MRI, and the arrow points to the area of the diffusion restriction. These changes represent acute ischemic infarction in the left posterior inferior cerebellar artery (PICA) territory without hemorrhagic transformation.

**FIGURE 5:**
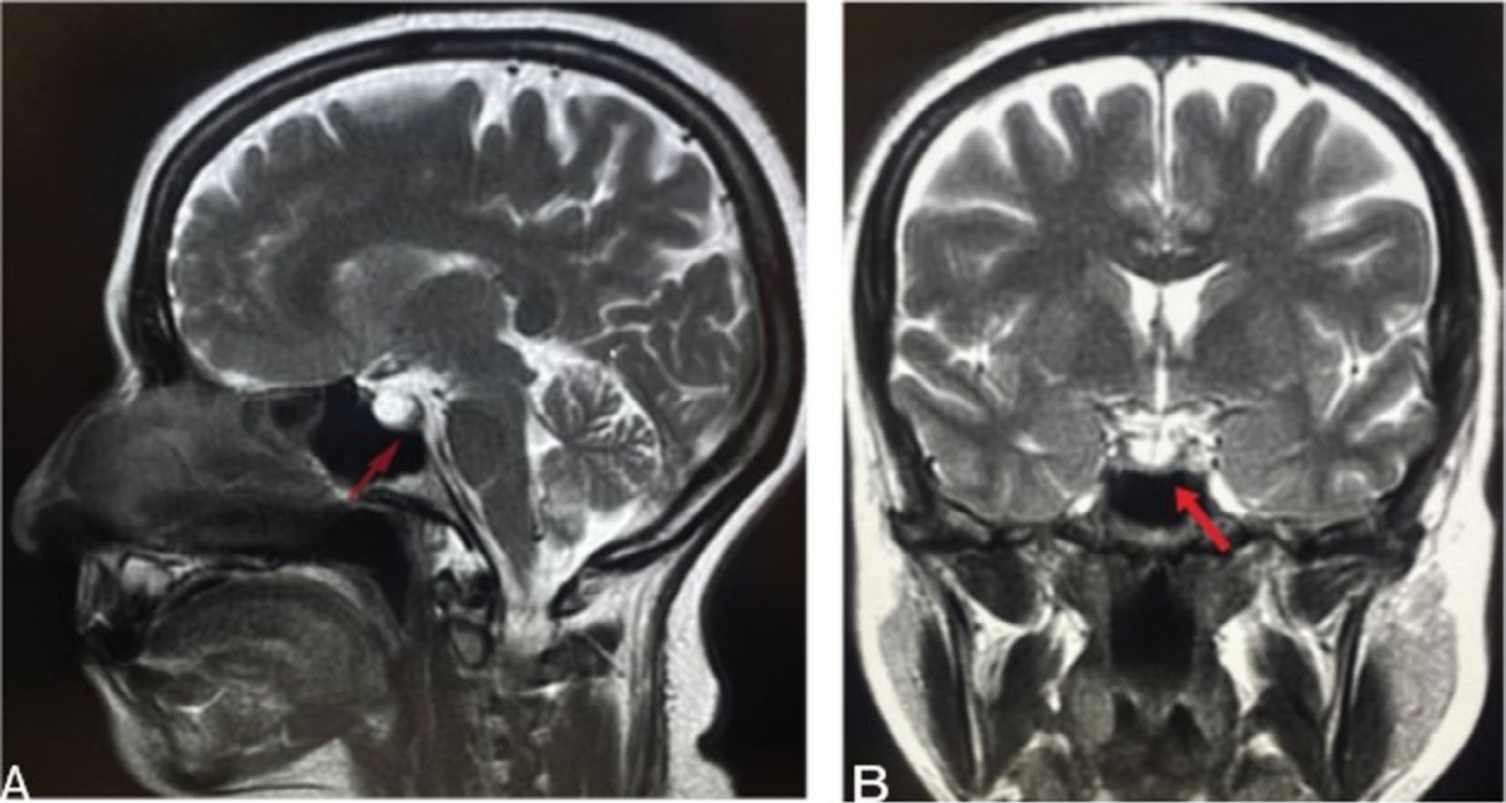
The arrows in both picture points to partial empty sella, (A) sagittal plane and (B) coronal plane. Partial empty sella may indicate various pathology, like elevated intracerebral pressure.

**FIGURE 6:**
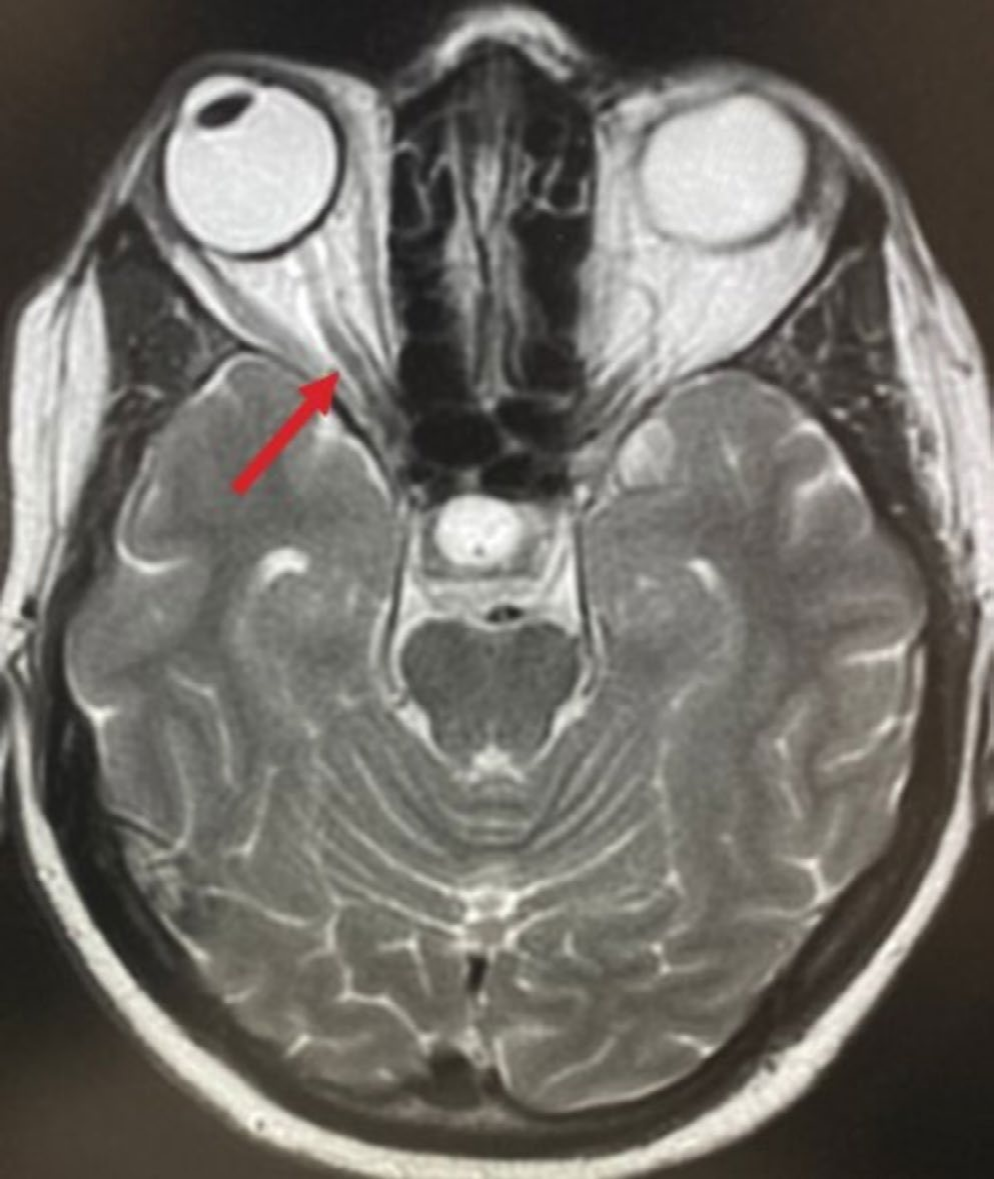
Shows a brain MRI image revealing with the red arrow pointing to the prominent perioptic subarachnoid space. This area contain CSF, and an enlargement may indicate elevated intracerebral pressure.

Vasculitis was at the top of our differential diagnosis; therefore, we ordered ANA and ANCA tests to confirm the diagnosis, which were negative for both. Therefore, vasculitis was excluded, and we started looking for other underlying causes. Additionally, the patient tested negative for HBsAg and anti‐HCV antibodies. Laboratory tests showed elevated inflammatory markers (ESR = 100 mm/hour, normally ≤ 20 mm/hour) and were positive for CRP (2.77 mg/dL, normally ≤ 0.5 mg/dL) (Table 1). Moreover, complete blood count (CBC) results were within the normal range except for mild elevation in the platelet counts, and creatinine levels were also normal. Coagulation test like PT, PTT, and INR where within normal ranges, and serum levels of protein‐C and anti‐thrombin III were also within normal range. However, platelet counts were slightly elevated about 473,000/μL (normal range 150 to 450 thousand/μL). CTA revealed partial filling defects in the proximal part (near the origin) of the left subclavian artery (Figure 2). The patient reported several episodes of fainting. Brain CT showed subtle hypodensities in the left cerebellar hemisphere (Figure 3). There were neither intra‐ nor extracranial hemorrhages with a normal ventricular system. Brain magnetic resonance imaging (MRI) was used to investigate hypodensities observed on CT. Brain MRI showed an area of abnormal signal intensity with diffusion restriction only in the inferior medial portion of the left cerebellar hemisphere, representing acute ischemic infarction in the left posterior inferior cerebellar artery (PICA) territory without hemorrhagic transformation (Figure 4). On post‐contrast images, grossly preserved enhancements of the vertebral, basilar, and proximal left PICA were observed. There was neither a midline shift nor a mass effect, with no vasogenic edema, the ventricular system was within the normal size, and a partial empty sella was evident (Figure 5). A prominent perioptic subarachnoid space (Figure 6) correlated with signs of increased intracranial pressure (ICP). Normal enhancement of the dural sinuses with no stenosis in the dominant right transverse sinus or thrombosis was observed. An echocardiogram was used to detect what gave rise to the partial filling defect in the left subclavian artery, it revealed mild left ventricular hypertrophy, mitral valve regurgitation grade 1, and trace tricuspid valve regurgitation.

**TABLE 1:** Laboratory findings.

	Value	Reference range
ESR	100	≤ 20 mm/hour
CRP	2.77	0–0.5 mg/dL
AST	13.0	42.0–54.5 (U/L
RBC	3.89	4.2–5.3 × 10^6^/μL
HB	11.6	11.7–16 g/dL
PCV	34.6	35%–47%
Platelet	473,000/μL	150–450,000/μL
Anti‐thrombin III	103.5%	80%–130%
Protein C	127.4%	70%–140%
Glucose	6.17	4.2–6.4 mmol/L
Creatinine	67	44–80 μmol/L
ANA	Negative	
ANCA	Negative	
Anti‐HCV	Negative	
HBsAg	Negative	

## 4 Treatment

Initially, the patient was treated with anticoagulant (Enoxaparin 80 mg/0.8 mL as Sc injection two times per day) and antiplatelet (aspirin 100 mg orally 1 time per day) drugs, and the digital ischemia stabilized (Figure 1). However, amputation was not performed because the digital ischemia was dry and was not complicated by infection.

## 5 Follow‐Up

At the 1‐month follow‐up, the patient was well and did not complain of any symptoms, and resolution of the digital infarction was evident.

### 5.1 Literature Review

A literature review was conducted using the PubMed database, limiting the search to the past 5 years. The following search terms were used: ((“digital ischemia”) NOT (“myocardial” OR “stroke” OR “intestinal” OR “mesenteric” OR “rectal” OR “subtraction”)). This yielded 134 studies; after meticulous exclusion of irrelevant studies, 60 studies were identified as relevant. The scope of the included studies encompassed digital ischemia or infarction regardless of whether they were idiopathic (Table 2). Despite this meticulous search strategy, no case reports specifically addressing idiopathic digital ischemia have been published.

**TABLE 2:** Characteristics of patients with digital infarction.

Study ID	DOP	Age (years)	Gender	Fingers infarcted	The disease underlying the infarction	Outcome
Attal et al, (2018) [5]	2018	83	Female	Left hand	Ranibizumab	Auto‐amputation of left fifth distal phalange and complete healing of other fingers
Martins‐Rocha et al, (2020) [6]	2018	14 months	Female	Right third and fourth	Post‐infectious	Complete resolution
Hari and Skeik, (2020) [7]	2019	52	Male	Bilateral second and right fourth	Behcet	Complete resolution
Kampoli et al, (2019) [8]	2019	78	Male	Right second–fifth, left third	Clear‐cell renal‐cell carcinoma ass paraneoplastic	Symptomatic improvement
Alzayer and Hasan, (2019) [9]	2019	39	Male	Left second and third	Hypereosinophilic vasculitis	Symptomatic improvement
Khaddour et al, (2019) [10]	2019	68	Female	Right first–fourth; left first second, third, and fifth	Immune checkpoint inhibitors	Progressed to dry gangrene
Zenati et al, (2020) [11]	2019	47	Male	Right second	Ipilimumab	Digital ulceration
Peña Arce et al, (2019) [12]	2019	45	Female	Bilateral second–fifth	HCV vasculitis	Dry gangrene in some fingers
Kurup and Simpson, (2019) [13]	2019	43	Male	All fingers	Sepsis	Complete resolution
Mülkoğlu and Genç, (2019) [14]	2019	37	Female	Right third–fifth	Hypothenar hammer syndrome	Complete resolution
Antonescu et al, (2019) [15]	2019	47	Female	All fingers	Fibromuscular dysplasia	Symptomatic improvement
St‐Pierre et al, (2019) [16]	2019	56	Male	Right third, fourth, fifth	Hypothenar hammer syndrome	Complete resolution
Schultz and Wolf, (2020) [17]	2020	Case 1: 70 Case 2: 43	Case 1: Female Case 2: Male	Case 1: Right 2nd‐4th Case 2: Right first and second	Case 1: Covid‐19 Case 2: Covid‐19	Case 1: Ischemia remained stable until she died Case 2: Eschar formation
Serra‐García et al, (2021) [18]	2020	48	Female	Right second–fourth; left fifth	Covid‐19	Symptomatic improvement
Cheemalavagu et al, (2020) [19]	2020	50	Female	Left second–fourth	Adalimumab‐induced antiphospholipid syndrome	Complete resolution
Aljahany et al, (2020) [20]	2020	26	Female	Right second	Epinephrine	Complete resolution
Kumar et al, (2020) [21]	2020	45	Female	Right first; left second	Brachial artery cannulation	Surgical amputation
Détriché et al, (2020) [22]	2020	31	Female	Left second–fifth	Arterial injection of crushed zolpidem	Surgical amputation of intermediate and distal phalanges
Imran et al, (2021) [23]	2020	30	Male	Right second	King cobra bite	Ray amputation
Ishii et al, (2020) [24]	2020	61	Female	Right third; left second	Hypereosinophilic syndrome	Complete resolution
Earl, (2020) [25]	2020	46	Female	Right second and third	Post transradial access	Surgical amputation
Martín Pedraz et al, (2022) [26]	2021	11	Male	Right fifth	Catastrophic antiphospholipid syndrome	Complete resolution
Shah et al, (2021) [27]	2021	34	Female	Right hand	Covid‐19	Progressed to dry gangrene, the patient died before the scheduled amputation
Collado et al, (2021) [28]	2021	48	Female	Right second and third; left second	Antiphospholipid syndrome and breast cancer	Necrosis increased until she died
Jesani et al, (2021) [29]	2021	57	Female	All fingers	Norepinephrine	Progressed to gangrene
Acherjee et al, (2021) [30]	2021	67	Male	Right first	Covid‐19	Symptomatic improvement
Vulasala et al, (2021) [31]	2021	51	Male	Right second; left second–fifth	Granulomatosis with polyangiitis causing Raynaud	Surgical amputation of three fingers
Jadhav et al, (2021) [32]	2021	18 weeks (26 weeks preterm)	Male	Right second	Septicemia	Complete resolution
Schjødt et al, (2021) [33]	2021	75	Male	All fingers	Covid‐19 + systemic sclerosis	Symptomatic improvement
Ravi et al, (2021) [34]	2021	45	Female	Left second–fifth	Undifferentiated connective tissue disease	Auto‐amputation of involved fingers
Klanidhi et al, (2021) [35]	2021	79	Female	Right second and fourth; left third and fourth	Acral vascular syndrome secondary to lymphoma	Gangrene
Rajiah et al, (2021) [36]	2021	32	Male	Right first	Adrenaline	Complete resolution
AlRasbi et al, (2021) [37]	2021	65	Male	Right second–fourth	Paraneoplastic acral vascular syndrome	Resolution of all fingers except third became gangrenous
Swarup et al, (2021) [38]	2021	58	Male	Right second	Necrotizing granulomatous vasculitis	Surgical distal amputation
Potluri et al, (2021) [39]	2021	56	Female	All fingers	Chemotherapy‐induced raynaud	Symptomatic improvement
Hong et al, (2021) [40]	2021	57	Female	Right fifth	Epinephrine containing nerve block	Surgical amputation
Chen et al, (2021) [41]	2021	52	Female	Left second–fifth	Malignancy associated antiphospholipid syndrome	Surgical amputation
Kennedy et al, (2022) [42]	2022	47	Female	Left fourth and fifth	Post transradial access	Surgical amputation of distal phalanges
Huang et al, (2022) [43]	2022				Oxaliplatin	
McNamara and Greyson, (2022) [44]	2022	72	Female	Right fourth	Raynaud + lidocaine and epinephrine injection	Wound healed with persistent stiffness
Patel et al, (2022) [45]	2022	41	Male	Left all fingers	Thenar hammer syndrome	Symptomatic improvement
Fuchsberger et al, (2022) [46]	2022	53	Female	Left first–third	Covid‐19	
Niitsuma et al, (2022) [47]	2022	70	Male	Right fifth	Blunt injury (hypothenar hammer syndrome)	Complete resolution
Estíbaliz et al, (2022) [48]	2022	63	Male	Right fourth	Hypothenar hammer syndrome	Complete resolution
Honan et al, (2022) [49]	2022	39	Female	Right second and third	Systemic sclerosis	Complete resolution
Akhlaghi Kalahroodi et al, (2022) [50]	2022	45	Male	Left second	ANCA vasculitis and antiphospholipid	Gangrene
Momen Majumder et al, (2022) [51]	2022	32	Male	All fingers	Non‐hodgkin lymphoma	Surgical amputation
Shoji et al, (2022) [52]	2022	66	Female	Left third	Systemic sclerosis	Complete resolution
Hidalgo Calleja et al, (2023) [53]	2023	60	Female	Right second–fifth	Graft versus host disease	Resolution for all fingers except third
Nayaz et al, (2023) [54]	2023	42	Female	Right first	Epinephrine	Complete resolution
Mateen et al, (2023) [55]	2023	52	Male	NR	Heparin induced thrombocytopenia	NR
Lee et al, (2023) [56]	2023	38	Female	Right third–fifth	Idiopathic radial artery occlusion	Symptomatic improvement
De Hous et al, (2023) [57]	2023	46	Female	Left fingers	Covid‐19	Unknown
Wangtiraumnuay et al, (2023) [58]	2023	19 months	Female	Left all fingers	Antiphospholipid + paraneoplastic + chemotherapy	Surgical amputation
Franco et al, (2023) [59]	2023	65	Female	All fingers	Systemic sclerosis + multiple myeloma	Surgical amputation
Dukan et al, (2023) [60]	2023	36	Male	Left second	Ruptured digital artery aneurysm	Complete resolution
Suwanto et al, (2023) [61]	2023	69	Male	Left first, third, and fourth	Dialysis access steal syndrome	Surgical amputation
Türkel et al, (2023) [62]	2023	54	Female	Left fifth	Gemcitabine	Surgical amputation of distal phalanx
Ibodeng et al, (2023) [63]	2023	41	Female	Left fourth and fifth	Hypothenar hammer syndrome	Complete resolution

Abbreviations: DOP, date of publication; NR, not reported.

## 6 Discussion

Digital infarction/ischemia is a rare condition with an incidence of 2 per 100,000 persons per year [64, 65], including cardiac or arterial embolism, systemic autoimmune connective tissue disorders (e.g., systemic sclerosis), thromboangiitis obliterans, vasculitis, iatrogenic (drug‐induced, especially chemotherapy [66, 67], or due to operations such as cannulation [21]), paraneoplastic acral vascular syndrome [68], local thrombosis, traumatic injury, and hypothenar hammer syndrome [2]. According to the literature review conducted for this study for all case reports of DI in the last 5 years, an emerging cause is COVID‐19 infection, with the cause of the DI being identified as a hypercoagulable state [69].

The definitive diagnosis of DI is primarily based on the clinical presentation of pain associated with permanent blanching or cyanosis of the digits, along with desquamation and ulceration. In our case, the patient exhibited some elements of this clinical picture, complaining of pain and bluish discoloration without desquamation or ulceration. This presentation led to the diagnosis of DI. However, establishing a specific etiology for our patient proved challenging.

Common causes of DI, such as cardioembolic disease and small vessel vasculitis, were ruled out through an echocardiogram that showed no thrombi in the heart and negative serum levels of ANCA, ANA, HBsAg, and anti‐HCV. A hypercoagulable state was excluded based on a normal coagulation profile, and the patient's non‐smoking status and uneventful obstetric history further supported this exclusion. Slightly elevated platelet counts were considered insignificant as platelet counts are elevated in stressful states, and elevated CRP and ESR supported this conclusion. Additionally, there was no clear history of Raynaud's phenomenon and on examination, there was no digital ulceration which mainly occurs due to the vascular involvement in scleroderma which excludes systemic sclerosis or scleroderma [70]. Moreover, there was no trauma to the affected hand or a medical or surgical history that could predispose to the condition.

Furthermore, the patient's free medical history and not remarkable physical examination along normal vitals led us to exclude paraneoplastic acral vascular syndrome. Consequently, the DI was labeled as idiopathic, given the absence of a specific underlying cause in the patient's clinical presentation.

The patient's high ESR titer and positive CRP align with findings in most cases of idiopathic DI, as reported by Raimbeau et al., where the mean CRP value in the idiopathic group was 28.4 ± 36.9, ranking as the second‐highest mean after the iatrogenic group. Despite this, the discovery is nonspecific, and there may be an alternative underlying etiology.

The treatment regimen involving anticoagulant and antiplatelet drugs in our case proved effective, leading to the patient's discharge without the necessity for more aggressive management options, such as Botox [71], or digital sympathectomy. The latter procedure is associated with long‐term benefits for patients experiencing digital ischemia due to autoimmune conditions [72]. With such a presentation and the absence of predisposing conditions or any significant findings, we present an idiopathic case of digital infarction.

However, diagnosing IDI should be established following excluding other possible causes, absence of the diagnosis should not prevent prompt conservative management like anticoagulant and antiplatelet therapy can prevent further tissue damage and improve patient outcomes. Clinicians should consider IDI in the differential diagnosis of patients presenting with digital ischemia, especially when common causes have been excluded.

In conclusion, idiopathic digital infarction poses diagnostic challenges owing to its rarity and lack of identifiable underlying causes. We present the first case report of IDI in a 47‐year‐old female of the three left lateral fingers. Thorough evaluation, including autoimmune serologies, echocardiogram, and coagulation profiles, ruled out most common causes, such as vasculitis, cardiac embolism, and hypercoagulable states. Imaging studies confirmed acute infarction in the left cerebellar hemisphere and proximal left subclavian artery. Despite the absence of a definitive etiology, the patient responded well to conservative management, emphasizing the importance of early diagnosis and comprehensive evaluation in such cases.

## Author Contributions

**Jaber H. Jaradat:** writing – original draft, writing – review and editing. **Wadi Walid I:** writing – original draft, writing – review and editing. **Aram F. Obeidat:** writing – original draft, writing – review and editing. **Raghad Amro:** writing – original draft, writing – review and editing. **Abdulqadir J. Nashwan:** writing – original draft, writing – review and editing.

## Consent

Written informed consent was obtained from the patient to publish this report, in accordance with the journal's patient consent policy.

## Conflicts of Interest

The authors declare no conflicts of interest.

## Data Availability

The data presented in this study are available within the article.
